# The Historical Speciation of *Mauremys* Sensu Lato: Ancestral Area Reconstruction and Interspecific Gene Flow Level Assessment Provide New Insights

**DOI:** 10.1371/journal.pone.0144711

**Published:** 2015-12-14

**Authors:** Huaxing Zhou, Yuan Jiang, Liuwang Nie, Huazong Yin, Haifeng Li, Xianmei Dong, Feifei Zhao, Huanhuan Zhang, Youguang Pu, Zhenfeng Huang, Jiaolian Song, Entao Sun

**Affiliations:** 1 The Provincial Key Lab of the Conservation and Exploitation Research of Biological Resources in Anhui, Anhui, China; 2 Life Science College, Anhui Normal University, Wuhu, China; 3 State Key Laboratory of Stem Cell and Reproductive Biology, Institute of Zoology, Chinese Academy of Sciences, Beijing, China; 4 Department of Medical Parasitology, Wannan Medical College, Wuhu, China; Trier University, GERMANY

## Abstract

*Mauremys* sensu lato was divided into *Mauremys*, *Chinemys*, *Ocadia*, and *Annamemys* based on earlier research on morphology. Phylogenetic research on this group has been controversial because of disagreements regarding taxonomy, and the historical speciation is still poorly understood. In this study, 32 individuals of eight species that are widely distributed in Eurasia were collected. The complete mitochondrial (mt) sequences of 14 individuals of eight species were sequenced. Phylogenetic relationships, interspecific divergence times, and ancestral area reconstructions were explored using mt genome data (10,854 bp). Subsequent interspecific gene flow level assessment was performed using five unlinked polymorphic microsatellite loci. The Bayesian and maximum likelihood analyses revealed a paraphyletic relationship among four old genera (*Mauremys*, *Annamemys*, *Chinemys*, and *Ocadia*) and suggested the four old genera should be merged into the genus (*Mauremys*). Ancestral area reconstruction and divergence time estimation suggested Southeast Asia may be the area of origin for the common ancestral species of this genus and genetic drift may have played a decisive role in species divergence due to the isolated event of a glacial age. However, *M*. *japonica* may have been speciated due to the creation of the island of Japan. The detection of extensive gene flow suggested no vicariance occurred between Asia and Southeast Asia. Inconsistent results between gene flow assessment and phylogenetic analysis revealed the hybrid origin of *M*. *mutica* (Southeast Asian). Here ancestral area reconstruction and interspecific gene flow level assessment were first used to explore species origins and evolution of *Mauremys* sensu lato, which provided new insights on this genus.

## Introduction

*Mauremys* sensu lato belonging to Geoemydidae was proposed as a new genus based on the recent molecular research. For this genus, nine species are generally recognized: *M*. *annamensis* from Vietnam, *M*. *caspica* from Turkey to Iran, *M*. *rivulata* from Northeast Mediterranean, *M*. *leprosa* from Southern Europe to Northern Africa, *M*. *mutica* from Southeast China and Vietnam, *M*. *reevesii* from China, Korea and Japan, *M*. *sinensis* from Southeast China, Laos and Vietnam, *M*. *nigricans* from Southern China, *M*. *japonica* from Japan [1].

In earlier studies using morphological comparisons, *Mauremys* sensu lato was divided into the narrow-jawed clade and broad-jawed clade based on the characteristics of the palate [2]. *Mauremys* sensu lato has been regarded to comprise four monophyletic groups; i.e., *Mauremys*, *Annamemys*, *Chinemys*, and *Ocadia* [3]. Subsequent studies based on molecular data firmly suggested the paraphyletic of four old genera [4–7]. Therefore, some authors have suggested combining these four monophyletic genera into an expanded genus; i.e., *Mauremys* sensu lato [1,5,6].

In recent years, more attention has been focused on the phylogeography and population genetic structure of *Mauremys* sensu lato. Previous studies of gene flow in this group mainly focused on Western Palearctic species. The existence of intraspecific gene flow was confirmed in *M*. *leprosa*, *M*. *caspica*, and *M*. *rivulata* [8–10]. Based on these results, Fritz *et al*. (2006) surmised the population of *M*. *leprosa* may be affected by glacial period bottlenecks, resulting in a decline in population diversity [9]. This inference has been confirmed in subsequent research about the population genetic structure of *M*. *japonica* [11]. However, the interspecific gene flow of East and Southeast Asian species has rarely been reported.

Eight species of *Mauremys* sensu lato were collected in this study. *M*. *nigricans* was not included because purebred *M*. *nigricans* is hardly found in the wild or turtle market. The phylogenetic relationships, interspecific divergence times, and ancestral area reconstruction of this group were explored using mt data. Subsequently, interspecific gene flow levels were assessed using five unlinked polymorphic microsatellite loci. Ancestral area reconstruction and interspecific gene flow level assessment were first used to explore species origins and evolution of *Mauremys* sensu lato, which provide new insights on the phylogeny of this genus.

## Materials and Methods

### 2.1 Ethics statement and Sample collection

Procedures involving animals and their care were consistent with NIH guidelines (NIH Pub. No. 85–23, revised 1996) and approved by the Animal Care and Use Committee of Anhui Normal University under approval number #20130710.

Thirty-two individuals of eight species included 18 living turtles and 14 specimens. No endangered or protected species were involved in this study. Twenty-five samples were collected from China and boundary areas adjacent to Vietnam. No permission was necessary for accessing areas where turtles were collected. All *M*. *japonica* and three West Asian species (*M*. *caspica*, *M*. *rivulata*, and *M*. *leprosa*) were purchased from the pet market; i.e., Yihe market in Guangdong (Table 1). Tissue samples were collected from the tails (3–5 mm from the tip) of living turtles at a sampling location using procedures that minimized pain. Before tissue collection, we used 5% lidocaine ointment to anesthetize the tails to alleviate pain and 70% alcohol to clean the tails to avoid infection. A low dose of antibiotic was applied on the wound after tissue collection. During the healing period, wound were kept dry.

**Table 1:** Listing of samples of *Mauremys* sensu lato.

Species	Original genus	Locality	Situation
***M*. *reevesii* 1**	*Chinemys*	Anhui, China	Specimen^*^
***M*. *reevesii* 2**	*Chinemys*	Anhui, China	Specimen^*^
***M*. *reevesii* 3**	*Chinemys*	Anhui, China	Specimen^*^
***M*. *reevesii* 4**	*Chinemys*	Anhui, China	Specimen^*^
***M*. *reevesii* 5**	*Chinemys*	Anhui, China	Specimen^*^
***M*. *annamensis* 1**	*Annamemys*	Guangxi, China (adjacent to Vietnam)	Specimen^*^
***M*. *annamensis* 2**	*Annamemys*	Guangxi, China (adjacent to Vietnam)	Specimen^*^
***M*. *annamensis* 3**	*Annamemys*	Guangxi, China (adjacent to Vietnam)	Specimen^*^
***M*. *mutica* (East Asian) 1**	*Mauremys*	Anhui, China	Specimen^*^
***M*. *mutica* (East Asian) 2**	*Mauremys*	Anhui, China	Specimen^*^
***M*. *mutica* (East Asian) 3**	*Mauremys*	Zhejiang, China	Live
***M*. *mutica* (East Asian) 4**	*Mauremys*	Zhejiang, China	Live
***M*. *mutica* (East Asian) 5**	*Mauremys*	Zhejiang, China	Live
***M*. *mutica* (East Asian) 6**	*Mauremys*	Zhejiang, China	Live
***M*. *mutica* (East Asian) 7**	*Mauremys*	Zhejiang, China	Live
***M*. *mutica* (Southeast Asian) 1**	*Mauremys*	Guangxi, China (adjacent to Vietnam)	Specimen^*^
***M*. *mutica* (Southeast Asian) 2**	*Mauremys*	Guangxi, China (adjacent to Vietnam)	Specimen^*^
***M*. *mutica* (Southeast Asian) 3**	*Mauremys*	Guangxi, China (adjacent to Vietnam)	Live
***M*. *mutica* (Southeast Asian) 4**	*Mauremys*	Guangxi, China (adjacent to Vietnam)	Live
***M*. *mutica* (Southeast Asian) 5**	*Mauremys*	Guangxi, China (adjacent to Vietnam)	Live
***M*. *mutica* (Southeast Asian) 6**	*Mauremys*	Guangxi, China (adjacent to Vietnam)	Live
***M*. *japonica* 1**	*Mauremys*	Japan	Specimen^*^
***M*. *japonica* 2**	*Mauremys*	Japan	Live
***M*. *japonica* 3**	*Mauremys*	Japan	Live
***M*. *japonica* 4**	*Mauremys*	Japan	Live
***M*. *sinensis* 1**	*Ocadia*	Guangdong, China	Live
***M*. *sinensis* 2**	*Ocadia*	Guangdong, China	Live
***M*. *sinensis* 3**	*Ocadia*	Guangdong, China	Live
***M*. *sinensis* 4**	*Ocadia*	Guangdong, China	Live
***M*. *rivulata***	*Mauremys*	Greece	live
***M*. *caspica***	*Mauremys*	Iran	Specimen^*^
***M*. *leprosa***	*Mauremys*	France	live

*All specimens were deposited in the provincial key laboratory of the conservation and exploitation research of biological resources in Anhui, China.

Most turtles were immediately released into the local habitat and others were fed in Anhui Normal University due to being an alien species. Specimens were deposited in the Provincial Key Laboratory of the Conservation and Exploitation Research of Biological Resources in Anhui, China. *M*. *mutica* were collected from two populations; i.e., an eastern China population and Vietnamese population. *M*. *mutica* from the two regions had obvious differences in morphology.

### 2.2 Laboratory protocols

Total genomic DNA was extracted from tail muscle tissue by a standard phenol/chloroform procedure via proteinase K digestion [12], and then kept at -20°C for PCR amplification.

Sixteen pairs of universal primers were designed for the mt DNA of *Mauremys* sensu lato (S1 Table). PCR reactions were conducted in 50 μL reaction mixtures containing 200 ng template DNA, 5 μL 10 × buffer (TaKaRa, Dalian, China), 4.0 μL MgCl_2_ (2.5 mol/L), 3.0 μL dNTP (2.5 mM), 2 μL of each primer (5 μmol/L), and 0.5 U Taq DNA polymerase (25 U/μL, TaKaRa). PCR conditions were as follows: initial denaturation (95°C, 1 min), then 35 cycles of denaturation (94°C, 50 s), primer annealing (50°C–58°C, 50 s), and elongation (72°C, 1 min) and a final extension (72°C, 10 min). The mt DNA fragments of intended sizes were recovered using a Gel Extract Purification Kit (TaKaRa). Purified PCR products were cloned into pMD19T vectors (TaKaRa) and all fragments were sequenced in both directions with an ABI3730 automated sequencer (Invitrogen Biotechnology Co., Ltd, USA).

Cross species microsatellite amplification was performed across 10 primer pairs developed for *M*. *reevesii* in earlier work of our laboratory (patent number: ZL201110026152.5) and five loci were chosen for amplification in this study. PCR conditions were as follows: 95°C for 5 min, 94°C for 30 s, 57°C for 60 s and 72°C for 90 s, followed by 32 cycles of step 2 to step 4 and final extension at 72°C for 5 min. SSR analysis was detected by ABIPRISM 3730. The results were read with GeneMarker software.

### 2.3 Genetic distance analysis

Complete mt sequences of 14 individuals of eight species (i.e., *M*. *mutica*, *M*. *japonica*, *M*. *sinensis*, *M*. *annamensis*, *M*. *reevesii*, *M*. *caspica*, *M*. *rivulata*, and *M*. *leprosa*) were sequenced. Twelve protein-coding genes [cytochrome *c* oxidase (COX) subunits 1, 2, and 3, cytochrome *b* (Cyt *b*), NADH dehydrogenase (ND) subunits 1, 2, 3, 4, 4L, and 5, and ATP synthase F0 (ATP) subunits 6 and 8] were chosen for analyses. Other genes [22 tRNA genes, 2 rRNA genes, a highly variable control region (CR) and ND6 gene] were excluded from the analyses due to potential saturation, alignment problems and different evolutionary rates, which influenced replacement patterns at the amino acid sequence level [13,14].

Twelve protein-coding genes were aligned separately with Mega 6.06 software [15] by ClustW (codon). Then, the genetic distances for all species were calculated by analysis of the combined data (10,854 bp) of mt 12 protein-coding genes using Mega v6.06.

### 2.4 Phylogenetic analysis

In order to resolve the current ambiguous phylogeny of *Mauremys* sensu lato, a total of 35 complete mt genome sequences of *Cuora* and *Mauremys* sensu lato, and out-group *Manouria emys* were selected (S2 Table).

Considering different evolutionary rates, twelve protein-coding genes were partitioned by codon positions and a best-fit substitution model was selected using PartitionFinder [16] (Table 2).

**Table 2:** Substitution models for nucleotide data partitions selected using the BIC in PartitionFinder.

Partition	Model
**ATP6, ATP8, Cytb, ND1, ND2, ND3, ND4L, ND4, ND5, CO1, CO2, CO3 1st and 2nd -codon**	**GTR+I+G**
**ATP6, ATP8, Cytb, ND1, ND2, ND3, ND4L, ND4, ND5, CO1, CO2, CO3 3rd-codon**	**HKY+I+G**

Phylogenetic relationships were inferred with maximum likelihood (ML) and Bayesian analysis (BI) under a best-fit partitioning scheme. ML tree was calculated with Raxml version 7.2.6 [17]. For each partition scheme, we performed a rapid (–f a -x option) with 1,000 replications to assess support on different nodes [17,18]. We regard bootstrap values of ≥ 70% as strong support and values of < 70% as weak support [19]. BI analysis was constructed using MrBayes v.3.1.2 [20]. BI was run with four Markov chains for 5 × 10^7^ generations and sampled every 1000 generations. The stationary point was reached when the potential scale reduction factor (PSRF) equaled 1, and when -log likelihood (-lnL) scores plotted against generation time reached a stationary value, and 25% of the generations were discarded. Trees from sample points following the burn-in were combined into a 50% majority rule consensus tree; the percentage of samples recovering a given clade reflected the clade’s posterior probability (PP). Posterior probabilities ≥ 95% are regarded as strong support.

### 2.5 Ancestral area reconstruction and divergence time estimation

Ancestral area reconstructions were inferred by the program RASP 3.0 [21] for speciational evolution in phylogenetic trees, using the Bayesian binary method (BBM) and the statistical dispersal-vicariance method (S-DIVA). All eight species were allocated to five areas where these turtles still existed: (1) East Asia (A); (2) Southeast Asia (B); (3) West Asia (C); (4) West Europe (D); (5) South Europe and North Africa (E).

The divergence times of these species have been estimated using BEAST 1.8.0 under a model of uncorrelated rates drawn from a log normal distribution for 1.5 × 10^8^ generations, assigning a Yule prior to rates of cladogenesis [22]. In order to ensure the time estimates to be as accurate as possible, all existing mt 12-protein sequences of Testudinidae, *Cuora* and *Mauremys* were selected from the NCBI database and *Pelomedusa subrufa* was used as the out-group (S3 Table). Two calibration points were selected to calibrate the divergence times: the fossil record of earliest divergence between Testudinidae and Geoemydidae, 55.0–66.4 million years ago (ma) [23]; the fossil record in Testudinidae, 45.6–55.8 ma [24].

### 2.6 Gene flow level assessment

Interspecific gene flow levels were assessed with Popgene 1.32 [25] using five unlinked polymorphic microsatellite data from 29 individuals except three Western Palearctic species (*M*. *rivulata*, *M*. *caspica* and *M*. *leprosa*). The interspecific genetic differentiation coefficients (Fsts) were calculated and 1,000 bootstrap resampling were carried out to verify confidence intervals (P < 0.05). Subsequently, the number of individual migrations in each generation was estimated by the effective number of migrants (Nm), which was calculated by the formula Nm = 0.25*(1-FST)/FST [26].

## Results

### 3.1 Characteristics of the mitochondrial gene data

Fourteen individuals of eight species were determined from 16,443 bp (GU938833 and NC_016951) to 17,067 bp (KP100055) in length. All complete mt genomes encoded for 2 rRNA, 22 tRNA, 13 protein-encoding genes and a highly variable control region (CR). Similar to other vertebrates, CRs of all species were located between tRNA^pro^ and tRNA^phe^. The following three parts of CR were also identified in these fourteen turtles: central conserved sequence block (CD), termination associated sequence (TAS), and conserved sequence blocks (CSB). And CSB contains a variable number of tandem repeat (VNTR) sequences. The arrangement of mt genes accorded with the general features of vertebrates.

Twelve protein-coding genes encoded by the H-strand were chosen for subsequent analyses because of the suitable evolutionary rate. The entire dataset was 10,854 bp in length, which contained 6,771 conserved sites, 4,080 variable sites, 3,072 parsim-informative sites and 1,008 singleton sites. The nucleotide compositions were slightly biased toward A and the average value was 32.3%.

### 3.2 Genetic distance comparative analyses between *Cuora* and *Mauremys* sensu lato

We calculated the genetic distance of *Cuora* and *Mauremys* sensu lato, respectively. Results for genetic distance showed that the largest genetic diversity in *Mauremys* sensu lato existed between *M*. *reevesii* and *M*. *leprosa* as well as *M*. *reevesii* and *M*. *annamensis* and the value was 0.086 (S4 Table). However, the largest value of *Cuora* was 0.093 and it existed between *C*. *mouhotii*, and *C*. *amboinensis* (S5 Table). Compared with *Cuora*, the genetic distance results suggested the largest divergence in *Mauremys* sensu lato was still at the species level within the genus.

### 3.3 Phylogenetic analyses

**Fig 1:**
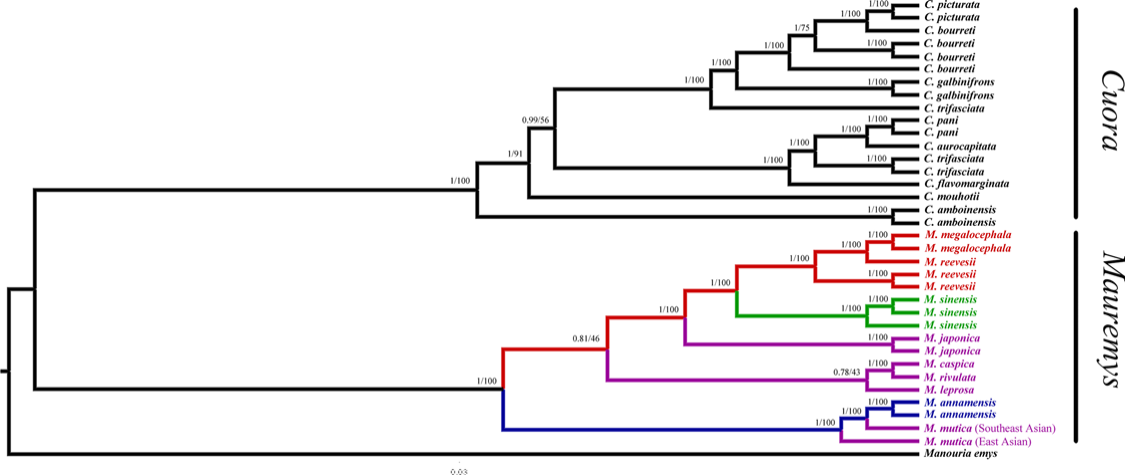
Phylogenetic trees for *Mauremys* sensu lato reconstructed based on mt heavy chain 12 protein-coding genes. Numbers of nearby branches are posterior probabilities (PPs, Left) and bootstrap proportions (BPs, Right) recovered from BI and ML analyses, respectively. Four old genera of *Mauremys* sensu lato are shown using different colours; i.e., red represents *Chinemys*; green represents *Ocadia*; purple represents *Mauremys*; blue represents *Annamemys*.

Phylogenetic relationships were estimated for the first time with ML and BI using mt twelve protein-coding genes. Trees derived from ML and BI analyses showed identical topology (Fig 1). Phylogenetic trees clearly indicated a sister relationship between *Cuora* and *Mauremys* sensu lato, while *Manouria emys* came out as the outgroup.

The "deeper" phylogenetic relationships in *Mauremys* sensu lato were divided into three clades (*M*. annamensis + *M*. *mutica* clade, *M*. *japonica* + *M*. *sinensis* + *M*. *reevesii* clade and *M*. *rivulata* + *M*. *caspica* + *M*. *leprosa* clade). The *M*. annamensis + *M*. *mutica* clade first diverged as the basal (PP = 1; BP = 100). The relationships between *M*. *rivulata* + *M*. *caspica* + *M*. *leprosa* and *M*. *japonica* + *M*. *sinensis* + *M*. *reevesii* and between *M*. *leprosa* and *M*. *rivulata* + *M*. *caspica* were not well supported (PP = 0.81; BP = 46 and PP = 0.78; BP = 43, respectively). However, the sister relationship between *M*. *rivulata* and *M*. *caspica* was strong supported (PP = 1; BP = 100) as well as *M*. *sinensis* + *M*. *reevesii* (PP = 1; BP = 100). *M*. *japonica* revealed a sister relationship with *M*. *sinensis* + *M*. *reevesii* with significantly statistical support (PP = 1; BP = 100).

In additon, the paraphyletic relationships between *M*. *reevesii* and *M*. *megalocephala* and between *M*. *annamensis* and *M*. *mutica* were revealed in both ML and BI trees (PP = 1; BP = 100 and PP = 1; BP = 100, respectively).

### 3.4 Divergence time estimation and ancestral area reconstruction

**Fig 2:**
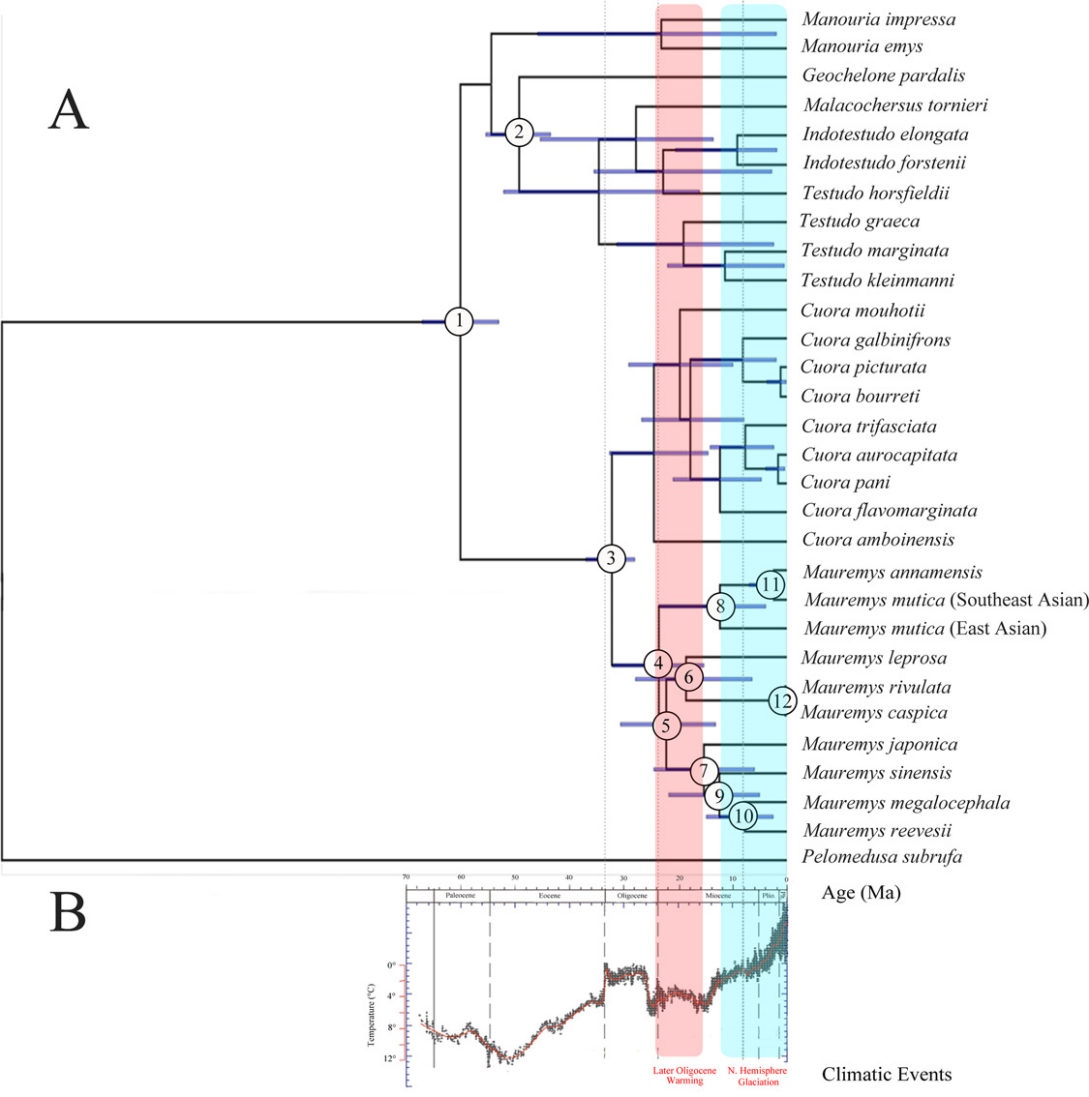
Chronogram using BEAST 1.8.0 based on mt heavy chain 12 protein-coding genes. A. Divergence time estimation; B. The trend of temperature change redrawn from Zachos et al.’s results [27]. The red zone represents the warming period. The blue zone represents the glacial period.

The results were read combining the trend of temperature change and climatic events (Fig 2) [27]. Divergence time estimation was calibrated by two fossil records (node 1 and node 2) [23,24]. The mean and 95% confidence interval (CI) of the ages of major nodes in the phylogeny are listed in Table 3. The divergence time between *Coura and Mauremys* sensu lato was consistent with Lourenco *et al*.’s results (mean: 32.26 ma with a 28.01–36.97 ma 95% CI) [24]. The earliest divergence of *Mauremys* sensu lato occurred in two Southeast Asian species (node 4; mean: 23.55 ma with a 12.26–32.12 ma 95% CI), and then species of the Eastern and Western Palearctic region diverged in 22.12 ma (node 5).

**Table 3:** Divergence time estimates in the chronogram shown in Fig 2.

Node	Fossil calibrated?	Lognormal priors	
		Mean (Ma)	95% CI
**1**	Yes	60.1	53.03–67.26
**2**	Yes	49.36	43.52–55.49
**3**	No	32.26	28.01–36.97
**4**	No	23.55	12.26–32.12
**5**	No	22.12	13.08–30.61
**6**	No	18.56	6.36–27.85
**7**	No	15.19	5.9–24.47
**8**	No	12.27	3.81–23.49
**9**	No	12.4	4.96–21.7
**10**	No	7.72	2.44–14.78
**11**	No	2.35	0.29–6.87
**12**	No	0.24	0.02–0.99

**Fig 3:**
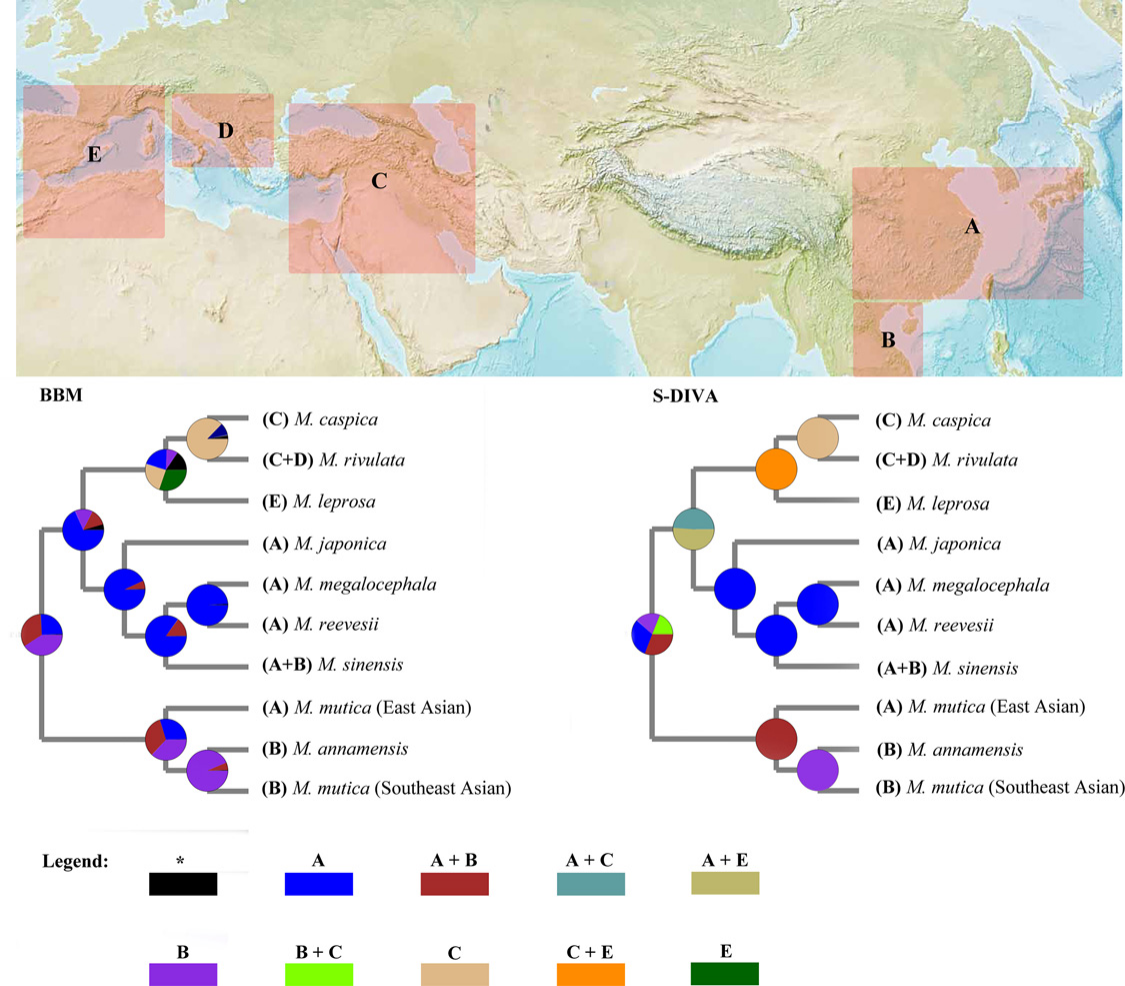
Results for ancestral area reconstruction inferred from BBM and S-DIVA based on mt heavy chain 12 protein-coding genes. The map is from the Central Intelligence Agency (CIA: https://www.cia.gov/library/publications/the-world-factbook/index.html). Potential original areas are coded as A: East Asia, B: Southeast Asia and Western Palearctic region (C: West Asia + D: West Europe + E: South Europe and North Africa), shown by different colours in the area pie chart.

The historical evolution of *Mauremys* sensu lato is clearly shown by the results of ancestral area reconstruction in Fig 3. The slight difference between the results of BBM and S-DIVA analyses were a consequence of assumptions underlying different methods. We preferred the results of BBM analysis compared to S-DIVA because BBM calculates the probability of each area based on the distribution of terminal taxa [21].

All results supported Southeast Asia (area B) as the original area of living members of *Mauremys* sensu lato and current distributions were formed by multiple diffusion. Diffusion began in Southeast Asia (area B), and then expanded step by step from East Asia (area A) to Western Palearctic region (areas C, D and E). Most species of *Mauremys* sensu lato were distributed in East and Southeast Asia, and only three species (*M*. *caspica*, *M*. *rivulata*, and *M*. *leprosa*) were distributed in Western Palearctic region.

### 3.5 The assessment of gene flow

Five microsatellite loci amplified unambiguous and repeatable products in the size range expected. We assessed the interspecific gene flow level of six populations of East and Southeast Asian species. The results are represented by Fst and Nm values (Table 4).

**Table 4:** Pairwise values of Fst (below diagonal) and Nm (above diagonal) among six populations of East and Southeast Asian species.

Fst \Nm*	1	2	3	4	5	6
**1. *M*. *annamensis***		0.96	1.14	1.64	1.34	1.10
**2. *M*. *reevesii***	0.21		1.02	1.28	1.01	1.34
**3. *M*. *japonica***	0.18	0.20		1.92	1.57	1.15
**4. *M*. *mutica* (Southeast Asian)**	0.13	0.16	0.12		4.81	2.63
***5*. *M*. *mutica* (East Asian)**	0.16	0.20	0.14	0.05		2.60
**6. *M*. *sinensis***	0.19	0.16	0.18	0.09	0.09	

*Nm = 0.25*(1-FST)/FST.

Strong gene flow existed among most all of the populations (Nm > 1), except for between *M*. *annamensis* and *M*. *reevesii* (Nm = 0.96). The maximum Nm and the minimum Fst occurred between the Southeast Asian population and East Asian population of *M*. *mutica* and this suggested no differentiation between the two populations of *M*. *mutica*.

## Discussion

### 4.1 Interspecific phylogenetic relationships of *Mauremys* sensu lato

In both ML and BI trees, species of old *Mauremys* (purple in Fig 1) were discovered in all three clades (*M*. annamensis + *M*. *mutica* clade, *M*. *japonica* + *M*. *sinensis* + *M*. *reevesii* clade and *M*. *rivulata* + *M*. *caspica* + *M*. *leprosa* clade). The paraphyletic relationship among four old genera; i.e., *Mauremys*, *Annamemys*, *Chinemys*, and *Ocadia*, was revealed clearly, so it suggested that the four old genera should be merged into the genus; i.e., *Mauremys*, which was consistent with previous studies based on different molecular data [4–7].

Compared with *Cuora*, the most diverse species in *Mauremys* sensu lato should remain in the same genus, which was clearly reflected in the genetic distance. The largest diversity recognized in *Cuora* was discovered between *C*. *mouhotii* and *C*. *amboinensis* and the value was 0.093. However, the interspecific maximum value in *Mauremys* sensu lato was 0.083 and this existed between *M*. *reevesii* and *M*. *leprosa* as well as *M*. *reevesii* and *M*. *annamensis*. The interspecific maximum genetic distance of *Mauremys* sensu lato was less than *Cuora*.

However, there is still controversy in recent molecular studies. Barth *et al*. (2004) and Spinks *et al*. (2004) suggested a sister relationship between *M*. *reevesii* and *M*. *japonica* + *M*. *sinensis* clade [4,6]. Also, Feldman *et al*. (2004) revealed *M*. *sinensis* was the sister to *M*. *japonica* + *M*. *reevesii* clade [5]. However, our results strongly supported a sister relationship between *M*. *japonica* and *M*. *sinensis* + *M*. *reevesii* clade.

Based on our phylogenies, an obvious paraphyletic relationship between *M*. *reevesii* and *M*. *megalocephala* was revealed. We inferred the reason for this paraphyletic relationship was that *M*. *megalocephala* is a “diet variant” of *M*. *reevesii*. This inference was in congruence with Barth *et al*.’s results [28].

### 4.2 The hypothesis for the origin and evolution of species in *Mauremys* sensu lato

Species of *Mauremys* sensu lato have a very wide distribution all over the Palearctic region. Some species are far apart and there is no gene exchange among them. However, they have an ultra-close phylogenetic relationship. This disjunction pattern had been reported for many species, e.g., softshell turtles, plants, fishes, amphibians, birds, and mammals [29,30].

We sampled extensively and traced the origins using mt genes. Combining divergence time and ancestral area reconstruction, we propose all species of *Mauremys* originated from a common ancestry in Southeast Asia. The earliest divergence of *Mauremys* occurred during the period of Late Oligocene Warming (25–16 Ma) (Fig 2, node 4). The warming climate provided a prerequisite for geographic radiations of *Mauremys*. Then, the divergence of species between the Eastern and Western Palearctic region was detected (Fig 2, node 5 and 6), while the turtles of *Mauremys* were diffusing from the East Asia to Western Palearctic region (Fig 3).

*M*. *japonica* diverged in approximately 15.19 Ma (Fig 2, node 7) while Japan was separating as an island arc by the Miocene back-arc opening of the Japan Sea during 15–25 Ma [31]. Considering that *M*. *japonica* is an endemic species in Japan, we suggest the divergence of this turtle may have been caused by the effects of the creation of the island of Japan.

The warm period broke in the late Miocene with the following glacial age [27] and turtles gathered in each refuge again. During long term evolution, genetic drift plays an important role that may lead to speciation [32]. Then permanent isolation between the species of *Mauremys* occurred by the emergence of deserts, oceans and mountains until now [33].

### 4.3 Interspecific gene flow level assessment for Southeast Asian and East Asian species

We assessed the interspecific gene flow level based on five unlinked polymorphic microsatellite loci. The results indicated the presence of extensive gene flow among East Asian and Southeast Asian species.

Interestingly, phylogenetic analyses based on mt genes showed obvious paraphyletic relationship between *M*. *annamensis* and *M*. *mutica*. The Southeast Asian population of *M*. *mutica* was closer to *M*. *annamensis* than the East Asian population of *M*. *mutica*. However, extremely extensive gene flow has been detected between the two populations of *M*. *mutica* and the value of Fst revealed that the two populations were hardly differentiated. The inconsistency in the two different data sets suggested the hybrd origin of Southeast Asian *M*. *mutica*. Compared with the study of Qi [34], we inferred sex-biased dispersal of *M*. *annamensis* may have occurred. The females of *M*. *annamensis* mated with the males of *M*. *mutica*. Then hybrids mated with *M*. *mutica*. The nuclear proportion of *M*. *annamensis* was diluted for multi-generations and mitochondria were preserved. Nevertheless, further evidence is required.

The extensive gene flow indicated the possibility of interspecific hybridization. More hybridization has been reported in *Mauremys* sensu lato, such as *O*. *glyphistoma* being a hybrid of *M*. *sinensis* and *M*. *cf*. *annamensis* [35] and *M*. *pritchardi* being hybrids of *M*. *reevesii* and *M*. *mutica*, respectively [36,37]. Therefore, the interspecific gene flow may have been increased by the turtle trade, escape from farms and release activities, which led to the genetic diversity of wild populations being lost.

## Supporting Information

S1 Fig Sampling sites and geographical coordinates in *Mauremys* sensu lato using DIVA-GIS. (TIF)

S1 Table Universal primers used to amplify complete mtDNA in *Mauremys* sensu lato. (DOCX)

S2 Table GenBank accession numbers of mitochondrial complete sequence of *Mauremys* sensu lato and *Cuora* for phylogenetic analysis. (DOCX)

S3 Table Thirty representative turtles used for divergence time estimate. (DOCX)

S4 Table Interspecific genetic distance of *Mauremys* sensu lato. (DOCX)

S5 Table Interspecific genetic distance of *Cuora*. (DOCX)
