# Supplementary figures and images for: The Historical Speciation of Mauremys Sensu Lato: Ancestral Area Reconstruction and Interspecific Gene Flow Level Assessment Provide New Insights
Source: PLoS One. 2015 Dec 14;10(12):e0144711. doi: 10.1371/journal.pone.0144711 (PMC4678219; doi:10.1371/journal.pone.0144711)

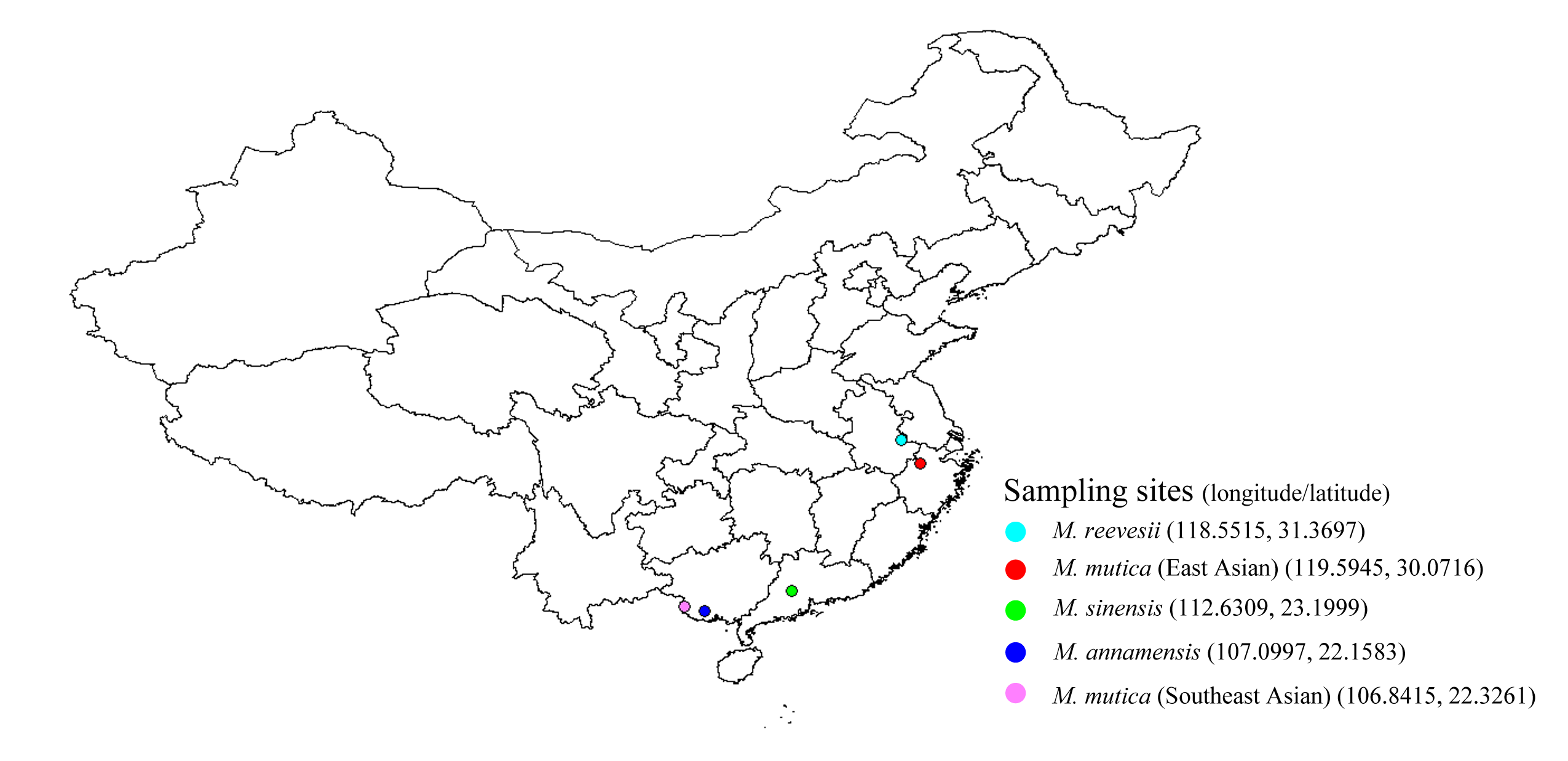

Supplement: S1 Fig — (TIF) [file pone.0144711.s001.tif]
