# Supplementary material for: The Historical Speciation of Mauremys Sensu Lato: Ancestral Area Reconstruction and Interspecific Gene Flow Level Assessment Provide New Insights
Source: PLoS One. 2015 Dec 14;10(12):e0144711. doi: 10.1371/journal.pone.0144711 (PMC4678219; doi:10.1371/journal.pone.0144711)
Supplement: S1 Table — (DOCX) [file pone.0144711.s002.docx]

**S1 Table. Universal primers used to amplify complete mtDNA in *Mauremys* sensu lato.**

| **Gene** | **Name** | **Sequences** | **Annealing temperature** |
| --- | --- | --- | --- |
| 12s rRNA | G1 | TTTCATGTTTCCTTGCGGTAC | **51-53℃** |
|  | G2 | AAAGCACGGCACTGA AGATGC |  |
| 16s rRNA | G3 | AAAGCATTCAGCTTACACCTGA | **52-55℃** |
|  | G4 | AAGTTCCACAGGGTCTTCTCG |  |
| 16s rRNA + ND1 | G5 | GTCTCTTACAAATAATCAGTGA | **50-53℃** |
|  | G6 | AGATTAGGTATATTGGTTCTTG |  |
| ND2 | G7 | ACCTGACAAAAACTAGCCCCA | **55-57℃** |
|  | G8 | ACTATTCCTGCTCAGGCHCCG |  |
| CO1 | G9 | THTTCTCYACYAACCAYAAAG | **52-55℃** |
|  | G10 | AAATCCTGCTATRATRGCGAA |  |
| ATP6, 8 | G11 | AAACAGACGCARTCCCAGGACG | **50-53℃** |
|  | G12 | GTTATTAGTAGTGCTGCTGYTGC |  |
| ND4L + ND4 | G13 | AGTACAAATGACTTCCAATCA | **56-58℃** |
|  | G14 | TTTGRTTWCCTCATCGTGTG |  |
| ND5 | G15 | AGGATAGAAGTAATCCAATGG | **50-53℃** |
|  | G16 | TATCTTTCGRATGTCTTGTTC |  |
| Cytb | G17 | AACCACCGTTGTATTCAACTA | **56-58℃** |
|  | G18 | CAATCTTTGGTTTACAAGACC |  |
| CR | G19 | GGAGGACAACCAGTAGAAAACCCA | **52-55℃** |
|  | G20 | ATTGGCTACACCTTGACCTGAC |  |
| ND1 + ND2 | G21 | TCCGGTTGAGCAGCTTCAAACTC | **50-53℃** |
|  | G22 | GTAGTTGGGTTTGGTTTAATCC |  |
| CO2 | G23 | GCTATCCCCAACAGGAGTAAAAG | **52-55℃** |
|  | G24 | GCTATCCTGTTTAGCTTCTATAG |  |
| CO3 | G25 | GCCGCTACCTACAAGAAAAC | **50-53℃** |
|  | G26 | GAARAATCGAATTGAGAATGG |  |
| CR | G27 | AGCAGCCTCCATCCTWTACTT | **52-55℃** |
|  | G28 | CAGTCTCATTGAGTYGGCAG |  |
| ND4 + ND5 | G29 | GAACCCCTATCACGAAAACG | **56-58℃** |
|  | G30 | GCTGTTTTTACGGCTGTTTTTG |  |
| ND5 + ND6 | G31 | CATACACGCMTTCTTYAAAGC | **52-55℃** |
|  | G32 | CTAATAGTGATCCGAAGTTTCAT |  |
