# Supplementary material for: The Historical Speciation of Mauremys Sensu Lato: Ancestral Area Reconstruction and Interspecific Gene Flow Level Assessment Provide New Insights
Source: PLoS One. 2015 Dec 14;10(12):e0144711. doi: 10.1371/journal.pone.0144711 (PMC4678219; doi:10.1371/journal.pone.0144711)
Supplement: S2 Table — (DOCX) [file pone.0144711.s003.docx]

**S2 Table. GenBank accession numbers of mitochondrial complete sequence of *Mauremys* sensu lato and *Cuora* for phylogenetic analysis**

| **Species** | **Accession number** | **Author** |
| --- | --- | --- |
| ***M. reevesii*** | FJ469674 | Jang,K.H. and Hwang,U.W. |
| ***M. reevesii*** | NC_016681 | Jang,K.H. and Hwang,U.W. |
| ***M. reevesii*** | NC_006082 | This study |
| ***M. megalocephala*** | HM_132059 | This study |
| ***M. megalocephala*** | NC_015101 | This study |
| ***M. sinensis*** | FJ871126 | This study |
| ***M. sinensis*** | NC_016685 | This study |
| ***M. sinensis*** | KC333650 | Fang,X. *et.al.* |
| ***M. japonica*** | GU938833 | This study |
| ***M. japonica*** | NC_016951 | This study |
| ***M. annamensis*** | HM131942 | This study |
| ***M. annamensis*** | NC_017875 | This study |
| ***M. mutica* (East Asian)** | NC_009330 | This study |
| ***M. mutica* (Southeast Asian)** | KP100056 | This study |
| ***M. leprosa*** | KP100055 | This study |
| ***M. rivulata*** | KP100054 | This study |
| ***M. caspica*** | KC692465 | This study |
| ***C. galbinifrons*** | EU809939 | Nie,L.W. and Zhang,Y.Y. |
| ***C. galbinifrons*** | NC_014102 | Nie,L.W. and Zhang,Y.Y. |
| ***C. picturata*** | JF712890 | Nie,L.W. and Wu,L.H. |
| ***C. picturata*** | NC_017878 | Nie,L.W. and Wu,L.H. |
| ***C. bourreti*** | JN980701 | Nie,L.W. and Wu,L.H. |
| ***C. bourreti*** | JN020145 | Nie,L.W. and Wu,L.H. |
| ***C. bourreti*** | NC_017885 | Nie,L.W. and Wu,L.H. |
| ***C. bourreti*** | JN865214 | Nie,L.W. and Wu,L.H. |
| ***C. trifasciata*** | KF724151 | Li,W., Zhu,X., Zhao,J. and Shi,Y. |
| ***C. trifasciata*** | KF574821 | Li,W., Zhu,X., Zhao,J. and Shi,Y. |
| ***C. trifasciata*** | NC_022857 | Li,W., Zhu,X., Zhao,J. and Shi,Y. |
| ***C. pani*** | GQ889364 | Nie,L.W., Bi,T.T. and Zhang,Y.Y. |
| ***C. pani*** | NC_014401 | Nie,L.W., Bi,T.T. and Zhang,Y.Y. |
| ***C. aurocapitata*** | NC_009509 | Nie,L., Pu,Y. and Peng,Q. |
| ***C. galbinifrons*** | NC_012054 | Nie,L.W. and Zhang,Y.Y. |
| ***C. mouhotii*** | NC_010973 | Zhang,L., Nie,L., Cao,C. and Zhan,Y. |
| ***C. amboinensis*** | FJ763736 | Nie,L.W. and Jing,W.X. |
| ***C. amboinensis*** | NC_014769 | Nie,L.W. and Jing,W.X. |
| ***Manouria emys*** | NC_007693 | Parham,J.F. *et.al.* |
