# Supplementary material for: The Historical Speciation of Mauremys Sensu Lato: Ancestral Area Reconstruction and Interspecific Gene Flow Level Assessment Provide New Insights
Source: PLoS One. 2015 Dec 14;10(12):e0144711. doi: 10.1371/journal.pone.0144711 (PMC4678219; doi:10.1371/journal.pone.0144711)
Supplement: S3 Table — (DOCX) [file pone.0144711.s004.docx]

**S3 Table. Thirty representative turtles used for divergence time estimate**

| **Family** | **genus** | **Species** | **Accession number** |
| --- | --- | --- | --- |
| **Pelomedusidae** | *Pelomedusa* | *P. subrufa* | NC_001947 |
| **Geoemydidae** | *Mauremys* | *M. annamensis* | NC_017875 |
|  |  | *M. caspica* | KC692465 |
|  |  | *M. japonica* | NC_016951 |
|  |  | *M. leprosa* | KP100055 |
|  |  | *M. mutica* (East Asian) | NC_009330 |
|  |  | *M. mutica* (Southeast Asian) | KP100056 |
|  |  | *M. megalocephala* | NC_015101 |
|  |  | *M. reevesii* | NC_006082 |
|  |  | *M. rivulata* | KP100054 |
|  |  | *M. sinensis* | NC_016685 |
|  | *Cuora* | *C. amboinensis* | NC_014769 |
|  |  | *C. aurocapitata* | NC_009509 |
|  |  | *C. bourreti* | NC_017885 |
|  |  | *C. flavomarginata* | NC_012054 |
|  |  | *C. galbinifrons* | NC_014102 |
|  |  | *C. mouhotii* | NC_010973 |
|  |  | *C. pani* | NC_014401 |
|  |  | *C. picturata* | NC_017878 |
|  |  | *C. trifasciata* | NC_022857 |
| **Testudinidae** | *Indotestudo* | *I. elongata* | NC_007695 |
|  |  | *I. forstenii* | NC_007696 |
|  | *Malacochersus* | *M. tornieri* | NC_007700 |
|  | *Testudo* | *T. kleinmanni* | NC_007699 |
|  |  | *T. graeca* | NC_007692 |
|  |  | *T. horsfieldii* | NC_007697 |
|  |  | *T. marginata* | NC_007698 |
|  | *Manouria* | *M. emys* | NC_007693 |
|  |  | *M. impressa* | NC_011815 |
|  | *Geochelone* | *G. pardalis* | NC_007694 |
