# Supplementary material for: The Historical Speciation of Mauremys Sensu Lato: Ancestral Area Reconstruction and Interspecific Gene Flow Level Assessment Provide New Insights
Source: PLoS One. 2015 Dec 14;10(12):e0144711. doi: 10.1371/journal.pone.0144711 (PMC4678219; doi:10.1371/journal.pone.0144711)
Supplement: S4 Table — (DOCX) [file pone.0144711.s005.docx]

**S4 Table. Interspecific genetic distance of *Mauremys* sensu lato**

|  | 1 | 2 | 3 | 4 | 5 | 6 | 7 | 8 | 9 | 10 | 11 | 12 | 13 | 14 | 15 | 16 | 17 |
| --- | --- | --- | --- | --- | --- | --- | --- | --- | --- | --- | --- | --- | --- | --- | --- | --- | --- |
| 1. *M. leprosa* |  |  |  |  |  |  |  |  |  |  |  |  |  |  |  |  |  |
| 2. *M. reevesii* | 0.082 |  |  |  |  |  |  |  |  |  |  |  |  |  |  |  |  |
| 3. *M. reevesii* | 0.082 | 0.000 |  |  |  |  |  |  |  |  |  |  |  |  |  |  |  |
| 4. *M. megalocephala* | 0.085 | 0.011 | 0.011 |  |  |  |  |  |  |  |  |  |  |  |  |  |  |
| 5. *M. megalocephala* | 0.085 | 0.011 | 0.011 | 0.000 |  |  |  |  |  |  |  |  |  |  |  |  |  |
| 6. *M. reevesii* | 0.086 | 0.010 | 0.010 | 0.014 | 0.014 |  |  |  |  |  |  |  |  |  |  |  |  |
| 7. *M. sinensis* | 0.082 | 0.047 | 0.047 | 0.051 | 0.051 | 0.053 |  |  |  |  |  |  |  |  |  |  |  |
| 8. *M. sinensis* | 0.082 | 0.047 | 0.047 | 0.051 | 0.051 | 0.053 | 0.000 |  |  |  |  |  |  |  |  |  |  |
| 9. *M. sinensis* | 0.081 | 0.035 | 0.035 | 0.038 | 0.038 | 0.040 | 0.013 | 0.013 |  |  |  |  |  |  |  |  |  |
| 10. *M. japonica* | 0.084 | 0.051 | 0.051 | 0.055 | 0.055 | 0.056 | 0.049 | 0.049 | 0.049 |  |  |  |  |  |  |  |  |
| 11. *M. japonica* | 0.084 | 0.051 | 0.051 | 0.055 | 0.055 | 0.056 | 0.049 | 0.049 | 0.049 | 0.000 |  |  |  |  |  |  |  |
| 12. *M. caspica* | 0.075 | 0.072 | 0.072 | 0.074 | 0.074 | 0.077 | 0.072 | 0.072 | 0.071 | 0.071 | 0.071 |  |  |  |  |  |  |
| 13. *M. rivulata* | 0.075 | 0.072 | 0.072 | 0.074 | 0.074 | 0.077 | 0.073 | 0.073 | 0.072 | 0.072 | 0.072 | 0.001 |  |  |  |  |  |
| 14. *M. annamensis* | 0.082 | 0.082 | 0.082 | 0.084 | 0.084 | 0.086 | 0.083 | 0.083 | 0.083 | 0.082 | 0.082 | 0.076 | 0.076 |  |  |  |  |
| 15. *M. annamensis* | 0.082 | 0.082 | 0.082 | 0.084 | 0.084 | 0.086 | 0.083 | 0.083 | 0.083 | 0.082 | 0.082 | 0.076 | 0.076 | 0.000 |  |  |  |
| 16. *M. mutica* (Southeast Asian) | 0.079 | 0.078 | 0.078 | 0.080 | 0.080 | 0.082 | 0.080 | 0.080 | 0.080 | 0.078 | 0.078 | 0.072 | 0.072 | 0.009 | 0.009 |  |  |
| 17. *M. mutica* (East Asian) | 0.082 | 0.077 | 0.077 | 0.079 | 0.079 | 0.081 | 0.079 | 0.079 | 0.078 | 0.080 | 0.080 | 0.076 | 0.077 | 0.058 | 0.058 | 0.057 |  |
