# Supplementary material for: The Historical Speciation of Mauremys Sensu Lato: Ancestral Area Reconstruction and Interspecific Gene Flow Level Assessment Provide New Insights
Source: PLoS One. 2015 Dec 14;10(12):e0144711. doi: 10.1371/journal.pone.0144711 (PMC4678219; doi:10.1371/journal.pone.0144711)
Supplement: S5 Table — (DOCX) [file pone.0144711.s006.docx]

**S5 Table. Interspecific genetic distance of *Cuora***

|  | 1 | 2 | 3 | 4 | 5 | 6 | 7 | 8 | 9 | 10 | 11 | 12 | 13 | 14 | 15 | 16 | 17 | 18 |
| --- | --- | --- | --- | --- | --- | --- | --- | --- | --- | --- | --- | --- | --- | --- | --- | --- | --- | --- |
| 1. *C. galbinifrons* |  |  |  |  |  |  |  |  |  |  |  |  |  |  |  |  |  |  |
| 2. *C. galbinifrons* | 0.000 |  |  |  |  |  |  |  |  |  |  |  |  |  |  |  |  |  |
| 3. *C. picturata* | 0.039 | 0.039 |  |  |  |  |  |  |  |  |  |  |  |  |  |  |  |  |
| 4. *C. picturata* | 0.039 | 0.039 | 0.000 |  |  |  |  |  |  |  |  |  |  |  |  |  |  |  |
| 5. *C. bourreti* | 0.039 | 0.039 | 0.001 | 0.001 |  |  |  |  |  |  |  |  |  |  |  |  |  |  |
| 6. *C. bourreti* | 0.038 | 0.038 | 0.006 | 0.006 | 0.005 |  |  |  |  |  |  |  |  |  |  |  |  |  |
| 7. *C. bourreti* | 0.038 | 0.038 | 0.006 | 0.006 | 0.005 | 0.000 |  |  |  |  |  |  |  |  |  |  |  |  |
| 8. *C. bourreti* | 0.038 | 0.038 | 0.009 | 0.009 | 0.009 | 0.006 | 0.006 |  |  |  |  |  |  |  |  |  |  |  |
| 9. *C. trifasciata* | 0.065 | 0.065 | 0.065 | 0.065 | 0.065 | 0.062 | 0.062 | 0.062 |  |  |  |  |  |  |  |  |  |  |
| 10. *C. pani* | 0.069 | 0.069 | 0.072 | 0.072 | 0.072 | 0.069 | 0.069 | 0.070 | 0.077 |  |  |  |  |  |  |  |  |  |
| 11. *C. pani* | 0.069 | 0.069 | 0.072 | 0.072 | 0.072 | 0.069 | 0.069 | 0.070 | 0.077 | 0.000 |  |  |  |  |  |  |  |  |
| 12. *C. aurocapitata* | 0.072 | 0.072 | 0.074 | 0.074 | 0.074 | 0.072 | 0.072 | 0.073 | 0.079 | 0.007 | 0.007 |  |  |  |  |  |  |  |
| 13. *C. trifasciata* | 0.069 | 0.069 | 0.070 | 0.070 | 0.070 | 0.068 | 0.068 | 0.069 | 0.076 | 0.032 | 0.032 | 0.036 |  |  |  |  |  |  |
| 14. *C. trifasciata* | 0.069 | 0.069 | 0.070 | 0.070 | 0.070 | 0.068 | 0.068 | 0.069 | 0.076 | 0.032 | 0.032 | 0.036 | 0.000 |  |  |  |  |  |
| 15. *C. flavomarginata* | 0.077 | 0.077 | 0.076 | 0.076 | 0.076 | 0.075 | 0.075 | 0.076 | 0.084 | 0.059 | 0.059 | 0.062 | 0.060 | 0.060 |  |  |  |  |
| 16. *C. mouhotii* | 0.071 | 0.071 | 0.073 | 0.073 | 0.073 | 0.072 | 0.072 | 0.072 | 0.079 | 0.075 | 0.075 | 0.077 | 0.074 | 0.074 | 0.080 |  |  |  |
| 17. *C. amboinensis* | 0.086 | 0.086 | 0.088 | 0.088 | 0.087 | 0.086 | 0.086 | 0.088 | 0.091 | 0.088 | 0.088 | 0.089 | 0.087 | 0.087 | 0.093 | 0.090 |  |  |
| 18. *C. amboinensis* | 0.086 | 0.086 | 0.088 | 0.088 | 0.087 | 0.086 | 0.086 | 0.088 | 0.091 | 0.088 | 0.088 | 0.089 | 0.087 | 0.087 | 0.093 | 0.090 | 0.000 |  |
